# Key considerations for designing, conducting and analysing a cluster randomized trial

**DOI:** 10.1093/ije/dyad064

**Published:** 2023-05-18

**Authors:** Karla Hemming, Monica Taljaard

**Affiliations:** Institute of Applied Health Research, University of Birmingham, Birmingham, UK; Clinical Epidemiology Program, Ottawa Hospital Research Institute, Ottawa, ON, Canada; School of Epidemiology, Public Health and Preventive Medicine, University of Ottawa, Ottawa, ON, Canada

**Keywords:** Cluster randomized trials, statistical analysis plans, cluster randomization justification, risk of bias

## Abstract

Not only do cluster randomized trials require a larger sample size than individually randomized trials, they also face many additional complexities. The potential for contamination is the most commonly used justification for using cluster randomization, but the risk of contamination should be carefully weighed against the more serious problem of questionable scientific validity in settings with post-randomization identification or recruitment of participants unblinded to the treatment allocation. In this paper we provide some simple guidelines to help researchers conduct cluster trials in a way that minimizes potential biases and maximizes statistical efficiency. The overarching theme of this guidance is that methods that apply to individually randomized trials rarely apply to cluster randomized trials. We recommend that cluster randomization be only used when necessary—balancing the benefits of cluster randomization with its increased risks of bias and increased sample size. Researchers should also randomize at the lowest possible level—balancing the risks of contamination with ensuring an adequate number of randomization units—as well as exploring other options for statistically efficient designs. Clustering should always be allowed for in the sample size calculation; and the use of restricted randomization (and adjustment in the analysis for covariates used in the randomization) should be considered. Where possible, participants should be recruited before randomizing clusters and, when recruiting (or identifying) participants post-randomization, recruiters should be masked to the allocation. In the analysis, the target of inference should align with the research question, and adjustment for clustering and small sample corrections should be used when the trial includes less than about 40 clusters.

Key Messages

- Cluster randomization should be used only when necessary: not only do cluster randomized trials require larger sample sizes than individually randomized trials, but they also have many other complexities.
- The potential for contamination is the most commonly used justification for using cluster randomization, but risks of contamination should be weighed against other risks before adopting cluster randomization.
- A key consideration with cluster randomization is the questionable scientific validity of inferences in settings with post-randomization identification or recruitment unblinded to the treatment allocation.
- When cluster randomization is necessary, adhering to key recommendations for this design is essential to minimize potential biases and maximize statistical efficiency.

## Introduction

In individually randomized trials, often referred to as patient randomized trials, individuals are allocated independently to different interventions, referred to here as treatment or control conditions. Instead of individuals, cluster randomized trials randomize entire clusters.^1^^,^^2^ Examples of clusters in a health care setting include wards, hospitals or primary care clinics and, in a non-health care setting, schools or communities.^3^ Cluster randomized trials are used to evaluate diverse types of interventions. Whereas clusters are randomized, the interventions may be delivered at the level of the cluster (e.g. a change to the electronic health care record), professionals within clusters (e.g. education of health care providers) or directly to individuals (e.g. a drug). For example, the CAP trial randomized primary care practices to evaluate an intervention delivered to the individual (cancer screen)^4^; whereas the CHIME-GP trial plans to randomize primary care practices to evaluate an educational intervention delivered to general practitioners.^5^ Moreover, many cluster trials evaluate complex interventions with components delivered at multiple levels.

Over and above the unit of intervention delivery, cluster randomized trials can have different levels for the unit of analysis. If the unit of randomization is different from the unit of analysis (e.g. medical practices are randomized and outcomes collected on individuals), observations are correlated within clusters, which reduces the effective sample size.^6^ Thus, cluster randomized trials require larger sample sizes compared with individually randomized trials.^7^^,^^8^ Cluster randomized trials can also be at greater risk of bias.^9–11^ Despite being at increased risk of bias and requiring larger sample sizes, cluster randomized trials are often selected for logistical reasons: simplifying the logistics of intervention delivery when everyone in the same cluster is treated in the same way.^12^ Cluster trials can also be advantageous when access to active interventions might be costly, or when there is a high risk of contamination.^12^ This design can also facilitate research embedded within usual care, particularly when it is ethically appropriate not to take individual-level consent and where routinely collected data are used for outcome assessment. Thus, cluster trials are also aligned with the pragmatic trials’ agenda—facilitating the evaluation of interventions in representative populations and under real-world conditions where there will be non-adherence, lack of compliance and co-interventions.^13^ Indeed, the use of cluster randomization has been steadily increasing over the past decades.^14^

However, cluster randomized trials are much more complex to design, analyse and report compared with individually randomized trials. Thus, although cluster randomized trials are an essential design in the toolkit of a health researcher, there are several critical requirements for their successful adoption, implementation and interpretation. Numerous systematic reviews have shown that there are major methodological concerns with published cluster randomized trials.^3^^,^^15–18^ In this manuscript, we provide 10 of the most important requirements for designing, conducting and analysing cluster randomized trials to help researchers overcome their major shortcomings (Box 1).

**Box 1:** The ten commandments for conducting a cluster randomized trial

1	The Golden Rule: methods that apply to individually randomized trials rarely apply to cluster randomized trials
2	Only use cluster randomization when necessary—balancing the benefits of cluster randomization with its increased risks of bias and increased sample size
3	Always randomize at the lowest possible level—balancing the risks of contamination with ensuring an adequate number of randomization units
4	Use the most statistically efficient designs
5	Always allow for clustering in the sample size calculation
6	Consider using restricted randomization and always adjust the analysis for covariates used in the restricted randomization
7	Always recruit participants before randomizing clusters whenever possible and when recruiting (or identifying) participants post-randomization, keep recruiters masked to the cluster allocation
8	Specify the target of inference to align with research questions
9	Always allow for clustering in the analysis
10	Always consider the use of a small sample correction in analysis

### 1. The golden rule: methods that apply to individually randomized trials rarely apply to cluster randomized trials

From planning to reporting, most methods that are used in individually randomized trials do not easily translate and apply to cluster randomized trials. For example, the types of interventions often evaluated in cluster trials, such as complex interventions and implementation strategies, can benefit from careful piloting and refinement before the definitive evaluation.^19^ Furthermore, when planning pilot or feasibility studies, there are many nuances to consider in cluster randomized trials—such as how many clusters are required to estimate an intra-cluster correlation coefficient (ICC) with reasonable stability^20^ and whether participants be recruited in a way that will not undermine the face validity of the trial.^9^ Likewise, these types of evaluations often benefit from a process evaluation or implementation evaluation alongside trial results, and so often require hybrid trial designs looking at effects on both clinical and implementation outcomes.^21^ When it comes to reporting, this should be according to the CONSORT extension to cluster randomized trials^22^ or its extension for stepped-wedge trials.^23^

### 2. Only use cluster randomization when necessary—balancing the benefits of cluster randomization with its increased risks of bias and increased sample size

Cluster randomization leads to an inflation in the required sample size and increases risk of bias compared with individually randomized trials. Exposing participants unnecessarily to the risks of research, either because the same question could have been answered with fewer participants or because the chosen design renders the results uninformative because of bias, is unethical.^22^ Because of this, adopting a cluster randomized design should always be carefully justified, and an individually randomized design used in preference wherever possible^12^—a piece of advice that has been around for some time.^24^

Cluster randomization is necessary when the intervention is delivered at the level of the cluster. Apart from this obvious justification, concerns over contamination (i.e. control participants inadvertently receiving the intervention) is one of the most common justifications for adopting a cluster randomized design.^3^^,^^12^ Interest in the total effect of an intervention (both its indirect and direct components) is another legitimate reason to use cluster randomization, arising particularly in vaccine trials: here, the contamination can be thought of as desirable.^12^ Likewise, opting to use cluster randomization in preference to individual randomization for logistical reasons or to avoid ‘disappointment’ effects (e.g. participants allocated to the control in an evaluation of a conditional cash transfer^25^) are other common reasons for adopting cluster randomization.^26^ Cluster randomization can also help with efforts to increase generalizability of findings—widening inclusion criteria and populations under evaluation.^27^ However, cluster randomization should never be adopted under the mistaken perception that it can help avoid seeking individual informed consent.^28^

Where participants have to be identified or recruited post-randomization, identification and recruitment bias operate in an unpredictable direction and can render a cluster trial much like an observational study (below).^11^ On the other hand, the impact of contamination is predictable: it will attenuate the treatment effect when those in the control inadvertently receive the intervention.^29^ If contamination can be accurately measured and an individually randomized approach adopted, it is possible to estimate the complier average causal effect (that is, estimating the impact among those complying with the treatment allocation). Furthermore, an adjustment can be made to the sample size calculation, and often the inflation to account for contamination is substantially less than the design effect due to clustering. Thus, the use of cluster randomization, particularly to protect against contamination, needs to be balanced against other risks: the use of cluster randomization can render the design at risk of unpredictable sources of bias (when it is used with unblinded post randomization recruitment), thus undermining its scientific validity.^29^

### 3. Always randomize at the lowest possible level—balancing the risks of contamination with ensuring an adequate number of randomization units

In some cluster randomized trials, there is a choice with respect to the unit of randomization, which can be at a higher or lower level.^8^ The choice involves a trade-off between logistics, contamination and statistical efficiency. An example of randomizing at a lower level is choosing to randomize wards rather than hospitals. Logistics are often enhanced when randomizing at a higher level. For example when randomizing hospitals, only one type of intervention is delivered throughout the hospital as opposed to in different wards. Contamination may also be reduced when randomizing at a higher level. For example, choosing to randomize hospitals rather than wards can prevent contamination due to providers in the same hospital being exposed to both treatment and control conditions. There are additional considerations when choosing clusters based on geographical areas: where clusters are too close in location, contamination might arise.

Nevertheless, statistical efficiency is increased when randomizing at a lower level: randomizing wards rather than hospitals could mean that more units are available for randomization. Statistical efficiency refers to the statistical power achieved from the available number of observations under the given design compared with the power that would have been available with the same number of observations under an alternative design. Power in a cluster trial is increased by increasing the number of clusters or increasing the cluster size. However, increasing the number of clusters leads to the largest increase in power (compared with increasing the cluster size), all other things being equal.^30^ When there is a choice to increase either the number of clusters or the cluster sizes, it is always better to increase the number of clusters.^30^

In theory, a parallel-arm cluster randomized trial can be implemented with a minimum of eight clusters (four per arm) which is the minimum required to obtain a *P-*value less than 0.05 under a randomization-based test.^26^ However, trials with a greater number of randomization units are much more likely to deliver on their wider objectives (will have increased face validity, will be amenable to an analysis based on standard approaches and are likely more generalizable) irrespective of their total sample size.^31^

### 4. Use the most statistically efficient design

Whereas increasing the number of clusters is the most statistically efficient way of increasing study power in theory, the number of clusters that can be included in practice is typically limited due to logistical or resource constraints.^32^ However, for a fixed number of clusters there are alternative ways of enhancing statistical efficiency, and this can be as simple as including a baseline measure of the response.^33^ In closed cohort designs, a baseline measure of the outcome could potentially be taken on all participants (ideally before randomization) so that the same participant is measured before and after randomization.^34^ In repeated cross-sectional designs, measurements may be taken on different groups of participants before and after randomization. The baseline period may be prospective or retrospective, or baseline measures may be available in summary form at the cluster level. Using such baseline measures in parallel-arm designs can have substantial power benefits^35^; in other settings, an unequal allocation of clusters to treatment arms might improve statistical efficiency.^32^

For a fixed number of clusters, the best way to improve statistical efficiency is to adopt a bi-directional, crossover design (i.e. a trial in which clusters switch in both directions, i.e. from an experimental to comparator and visa versa^36^^,^^37^). In practice however, interventions evaluated in a typical cluster randomized trial are often not possible to withdraw. Where only unidirectional crossover is possible, adding control periods—either by using a parallel cluster randomized trial with baseline measures or extending this to a stepped-wedge cluster randomized trial—can increase efficiency, with gains depending on the intra-cluster correlations (the higher the correlations and larger the cluster sizes, the greater the rewards).^35^^,^^38^^,^^39^ As with cluster randomization, the risks and rewards of these alternative designs have to be balanced.^40^^,^^41^ For example, cluster crossover designs should not be used where there are risks of carry-over effects; and stepped-wedge designs might make assumptions about underlying secular trends and about ‘light switch’ intervention effects (i.e. that the intervention has an immediate and sustained impact).^42^^,^^43^

Of note, large cluster sizes can be statistically inefficient, especially when the intra-cluster correlation coefficient is high.^30^ This can mean that many observations within a cluster might not make a material contribution to the study power, and this can have implications for both the duration of the study and ethics (it might increase the duration or expose individuals to research risks for no material return).^44^ Thus, trialists should consider the implications of decreasing the sizes of clusters on study power as a way of determining if all cluster members are making a material contribution.

### 5. Always allow for clustering in the sample size calculation

The design and analysis of individually randomized trials assume independence of observations. Observations within clusters tend to be correlated, and this correlation is measured by the intra-cluster correlation coefficient (ICC). When treatment assignment depends on the cluster, as it does in a cluster randomized trial, clustering is said to be ‘informative’–in other words, it needs proper consideration in the sample size and analysis.^45^ Even when the anticipated ICC is low, the impact on the required sample size can still be important, particularly when the cluster sizes are large.^15^

Sample size estimation should ideally be based on the proposed analysis model (below). In most settings, this can be achieved by inflating the number needed under individual randomization by a ‘design effect’.^46^^,^^47^ Design effects are available for parallel cluster designs, cluster crossover designs, cluster designs with baseline periods and stepped-wedge designs (known as multiple period designs).^42^^,^^48^^,^^49^ Care is needed when using these design effect approaches for small samples or rare or common binary outcomes—and in these settings determining power by simulation might be warranted^50^ or extra clusters be added to each arm to accommodate the use of the t-distribution at the analysis stage (see Commandment 10^26^). Care is also needed in the setting where variation in the cluster sizes is anticipated—again increasing the number of clusters by 25% can protect against this.^51^

These approaches require the specification of measures of correlation, such as the ICC in a parallel design (more extensive correlations are required for multiple period designs), and sensitivity to assumptions about correlations should be investigated.^52^ When outcome data are available for a similar setting (perhaps a similar trial or routinely collected data source), correlations can be estimated to inform these calculations; more commonly, researchers have to be guided by likely values considering patterns reported in the literature.^53–55^ For binary outcomes, these correlations are needed on the proportions scale and not the logistic scale,^56^ and are also known to be dependent on the prevalence of the outcome.^57^^,^^58^

### 6. Consider using restricted randomization and always adjust the analysis for covariates used in the restricted randomization

Cluster trials often randomize a small number of units—substantially fewer than in typical individually randomized trials, and most use some form of restricted randomization.^3^^,^^59^ Restricted randomization methods can enhance the credibility of the trial results by protecting against imbalances in cluster and participant characteristics (sometimes referred to as enhancing face validity) and can also improve statistical power.^60^ These benefits are likely to be greater with a smaller number of randomization units (where the risk of chance imbalance will be larger).^61^ Restricted randomization methods use either cluster-level characteristics or cluster-level summaries of individual-level characteristics (e.g. cluster-level mean of primary outcome from a baseline period) to protect against poorly balanced allocations. There is limited guidance on the choice of factors for inclusion in a restricted randomization procedure, but as with covariate adjustment, factors should be chosen on the basis of their prognostic strength, availability and reliability. Examples of common restriction factors in cluster trials include cluster location, cluster size and a baseline measure of the outcome.^16^ Unlike individually randomized trials, where the randomization is usually implemented sequentially, in cluster trials, randomization of clusters is frequently implemented once-off or in batches.

There is a number of different approaches for restricted randomization in cluster trials, including stratified block randomization, minimization, covariate constrained randomization and pair matching.^59^ The appropriate method in any given trial often depends on logistical constraints in the setting. For example, minimization allows for sequential randomization, whereas covariate constrained randomization does not. Minimization might be preferred over stratification when there are many covariates to balance.^62^^,^^63^ Unlike stratification, both minimization and covariate-constrained randomization can balance on continuous covariates and so do not require categorization of prognostic factors.^64^ Pair matching has an intuitive appeal, but others have confirmed that its usefulness might be less than expected.^65^ Blocking can help prevent large imbalances in numbers of clusters allocated to each arm.^66^

It can be tempting to include many covariates in restricted randomization, but this can be problematic, for example leading to incomplete blocks in stratification^62^^,^^67^ or overly constrained designs where some pairs of clusters are always allocated to the same arm.^68^^,^^69^ Furthermore, when the allocation becomes more deterministic it becomes reliant on covariates being truly prognostic and can also lead to subversion of the allocation process where it is easy to predict upcoming allocations. Finally, when restricted randomization has been used, the analysis should adjust for the covariates used in the randomization to ensure nominal type I errors.^61^

### 7. Always recruit participants before randomizing clusters whenever possible, and when recruiting (or identifying) participants post-randomization, keep recruiters masked to the cluster allocation

To ensure allocation concealment, there is a recommended process which, in individually randomized trials, is almost always followed: individuals are recruited, and once their participation is ratified, they are randomized to the treatment or control condition.^70^ In cluster trials, this may be possible in closed cohort designs: for example, in a study where eligible participants are children within a school classroom. Indeed, the majority of cluster trials within a school setting use the closed cohort design.^71^ Alternatively, clusters are randomized and participants recruited continuously throughout the trial, with each participant providing a single measurement (known as a ‘continuous recruitment’ design). Continuous recruitment designs are used when eligible participants are incident populations, for example, if participants are people with a new diagnosis of hypertension, it would not be possible to recruit all participants before clusters were randomized. This reversal of the ordering can prevent cluster allocation being concealed from recruiters and participants at the time of recruitment, and this can be an important source of bias in cluster trials.^9^^,^^72^ There are numerous ways this bias can manifest—for example, recruiters and participants in the control arm might differentially recruit and agree to participation in the study compared with those in the intervention arm.^73^

Although it is preferable to recruit participants before randomization of clusters, in practice, this is not always possible. Sometimes cluster trials can be conducted without participant recruitment. For example, cluster randomized trials might involve whole clusters, i.e. a complete enumeration of the entire cluster of eligible participants. This may be appropriate when individuals are not considered research participants and when there are routinely available outcome data (e.g. mortality) and an ethics committee has granted a waiver of participant informed consent.^28^ In these settings, the risk of recruitment bias is remove but there remains the possibility that participants could be differentially identified across the study arms. Identification biases are a possible cause of the differential characteristics of participants across study arms in an unblinded evaluation of rapid screening for group B streptococcus in pregnancy using cluster randomization (no individual participant recruitment).^74^

When post-randomization recruitment or identification of participants is unavoidable, there will be a risk of recruitment biases, unless treatment conditions are blinded. However, due to the nature of interventions evaluated in cluster trials, blinding is often not possible. In these settings, mitigation strategies can help prevent recruitment biases.^9^^,^^72^ First, recruitment by someone independent of the trial who is blind to the cluster status will minimize risks of recruitment bias. Second, recruitment strategies should be consistent across the study arms—for example, consent forms should be similar under treatment and control conditions. Finally, where possible, keeping information on the precise details of the intervention only known to essential people can help.

Reporting whether participants were recruited post-randomization and whether recruiters and participants were aware of their allocation can help others identify these risks when interpreting trial results. Recommended reporting practices include clear reporting of blinding status along the timeline of recruitment and randomization.^75^ In addition, making the consent forms available improves transparency on what information was communicated to participants about the trial (e.g. were participants aware that their arm was the active intervention). In some settings there may be grounds for statistical testing of differences across intervention arms.^76^

### 8. Specify the target of inference to align with research questions

In individually randomized trials, the target of inference is almost always the individual, i.e. the trial objective is to determine the impact of the intervention on the typical individual. In cluster randomized trials, the trial objective might be to determine the impact of the intervention on either the typical individual or the typical cluster.^26^^,^^77^ Careful specification of the estimand (target of inference) helps identify the appropriate design and analysis.^78^ For example, cohort sampling aligns with an interest in the impact on the typical individual; and repeated cross-sectional sampling aligns with an interest in the impact on the typical cluster.^34^^,^^77^ Furthermore, depending on whether the target of inference is the typical cluster or typical individual, in conjunction with the possibility that the impact of the intervention might vary with cluster size (known as ‘informative cluster sizes’), an analysis approach should be chosen that appropriately targets the effect for the typical individual or the typical cluster (below).^79^^,^^80^

Other considerations when specifying the estimand include, but are not limited to, the type of summary measure and covariate adjustment. Whereas there is some debate over the best summary measure to report, it is likely that both an absolute and a relative measure will be appropriate in most trials.^22^ Direct covariate adjustment changes the target of inference from unconditional (the expected treatment effect for a typical individual, also known as the marginal effect) to a conditional estimate (the expected treatment effect for a particular subgroup of individuals defined by the covariates in the model).^81^ Differences will exist between these two estimands when the summary measure is non-collapsible (odds ratios and hazard ratios). Differences will be more noticeable when the outcome prevalence varies across sub-groups. Furthermore, if effects vary across sub-groups (effect modification), a conditional estimate might not be very useful.^81^ Marginal standardization, as an alternative to direct covariate adjustment, can be used to estimate the marginal covariate-adjusted summary measure, as too can inverse probability weighting.^82^^,^^83^

Any covariates to be included in a covariate-adjusted estimate of the treatment effect should be pre-specified and should include all covariates used in any restricted randomization.^16^^,^^61^ Unlike in individually randomized trials, adjustment for covariates might not always increase statistical precision.^84^ Adjusting for both cluster-level and individual-level versions (of the same covariate) can help capture residual confounding.^85^ Covariate adjustment is likely to be particularly important when there is post-randomization unblinded recruitment or identification of participants.^82^ Whereas care is needed in small samples, small sample corrections can maintain nominal type I errors alongside covariate adjustment.^61^^,^^82^ When adjusting for covariates in the context of low or high prevalence binary outcomes, model convergence can be problematic, and in these settings, propensity score approaches might help.^82^^,^^83^ Where covariate data are incomplete, any multiple imputation procedures should appropriately allow for the clustered nature of the design.^86^

### 9. Always allow for clustering in the analysis

Clustering should always be allowed for in the statistical analysis. Significance testing should not be used to compare models that do and do not allow for clustering. There are a number of ways to allow for the non-independence of observations in the analysis.^87^^,^^88^ The simplest is to carry out what is known as a cluster-level analysis.^89^ Essentially this consists of aggregating cluster-level outcomes by a summary statistic (such as the mean or proportion) and then using conventional analysis methods on these summary statistics (e.g. a t test^90^). For binary outcomes, this approach can be used in conjunction with transformations (e.g. log to report relative risks or logit to report odds ratios). Cluster-level approaches (unweighted by cluster size) allow inferences targeted at estimating the effect of the treatment for the typical cluster.^79^ When the objective is to determine the impact on the typical individual, this approach might not work well when there is substantial variation in cluster sizes.^91^ Furthermore, the cluster-level approach is more complex when it is desirable to adjust for individual-level covariates,^2^ which may explain why cluster-level approaches are infrequently used.^3^ However, cluster-level approaches are robust with a small number of clusters.^92^

If an individual-level analysis is preferred, two main approaches to accommodating the non-independence are generalized linear mixed models (GLMM) and generalized estimating equations (GEE).^87^ For odds ratios (and other non-collapsible link functions), these two approaches yield different interpretations of the treatment effect; in particular, GLMM yields a cluster-specific estimate, i.e. the effect of the treatment conditional on cluster membership, whereas GEE yields an unconditional (marginal) estimate. These approaches facilitate adjustment for individual-level covariates, but do not work well when there are fewer than about 50 clusters (40 for GLMMs), unless combined with a small sample correction (below).^92^ For continuous outcomes, these models typically have reasonable convergence properties, but when using binomial distributions and log or identity links for binary outcomes, convergence problems can arise (especially with rare or common outcomes). In these settings, modified robust Poisson models can be used to facilitate the estimation of relative risks.^93^ Although marginal standardization is a potential alternative option to estimate relative risks and risk differences, its performance has yet to be evaluated in cluster trials. Care is needed when using individual-level methods of analysis in settings where informative cluster size is considered plausible (here, GEE with independent estimating equations might be necessary).^79^ For multiple period designs, non-exchangeable correlations should be allowed for to avoid overestimation of statistical precision.^94^ For cluster trials with baseline periods, this can be accommodated using either a constrained baseline analysis with random cluster by period effects, or analysis of covariance (ANCOVA)-type approaches (adjusting for baseline values of the outcome—possibly at the level of the cluster).^95^

### 10. Always consider the use of a small sample correction in analysis

In what was probably the first methodological paper on cluster randomized trials, Cornfield in 1978 referred to two penalties to cluster randomization^6^: variance inflation due to clustering, which is a function of the ICC (also known as the ‘design effect’) and the degree of freedom penalty, often referred to as a small sample correction. The first of these is widely recognized as an implication of cluster randomization. The second turns out to be consequential but much less widely appreciated.^96^ The degree of freedom penalty is essentially a penalty to account for the use of a large-sample approximation (i.e. z test) when a more exact method is required, such as a permutation test or t test.^26^^,^^77^ It turns out that t tests are required when there are less than about 40 independent observations.^6^ When a t test is used, the degrees of freedom must be specified. Under individual randomization, the degrees of freedom for parallel-arm assignment are 2 N - 2, where N is the number of participants per arm.

Appropriate degrees of freedom in parallel cluster trials are not so clear-cut. Options include 2K - 2 (known as the ‘between-within’ correction), where K is the number of clusters per arm.^92^ This tends to work well for both continuous and binary outcomes when there is limited cluster size variation.^92^^,^^97^ Other available options are more complicated but might have better statistical properties (i.e. more likely to maintain type I error at 5%). For GLMMs, these degrees of freedom options include Kenward–Roger and Satterthwaite approximations.^97^^,^^98^ For GEEs, they include but are not limited to Kauermann–Carroll and Mancl–DeRouen.^99^ The performance of these is dependent on setting (e.g. number of clusters, cluster sizes, ICC, cluster size variation, outcome prevalence), and none appear to work well across the board. Typically, these ‘corrections’ estimate degrees of freedom to be used under a t test, and some of them also consist of making a ‘correction’ to the standard error of the treatment effect. Indeed, in addition to these degree of freedom corrections, robust standard errors (‘sandwich’ variance) or restricted maximum likelihood (REML) procedures should be used when fitting GEE or GLMM, respectively.^100^ Since cluster randomized trials typically include less than 40 clusters, almost all cluster trials should make allowance for this correction.^3^

## Conclusion

Cluster randomized trials face many complexities. Not only do they require larger sample sizes than individually randomized trials, especially for large cluster sizes and large intra-class correlations; other complexities include sometimes being more complicated to implement, being more vulnerable to poor sample size estimation and more vulnerable to invalidating model-based assumptions. Perhaps of most importance is their questionable scientific validity in settings with post-randomization identification or recruitment unblinded to the treatment allocation. The potential for contamination is the most commonly used justification for using cluster randomization, whereas the risks of identification and recruitment biases are less widely appreciated. This needs a shift in balance—and researchers should weigh up the different risks before adopting cluster randomization. Cluster randomization is essential when evaluating cluster-level interventions, but determining the level of the intervention is not always straightforward. When cluster randomization is necessary, these simple commandments should help researchers conduct these evaluations in a way that minimizes potential biases and maximizes statistical efficiency.

## Ethics approval

Not applicable.

## Data Availability

No new data were generated or analysed in support of this research.
